# Making the Unfit into Serviceable Recruits

**Published:** 1916-01-29

**Authors:** 


### GUY'S HOSPITAL AND THE WAR.

### Making the Unfit into Serviceable Recruits.

A question which has no doubt often occurred to that large section of the thinking public which interests itself in the care of the sick poor is whether the encroachment of the military authorities upon the bed accommodation of voluntary hospitals is causing any considerable amount of hardship among the civil population. It is common knowledge that nearly all the large London hospitals have undertaken the treatment of sick and wounded soldiers; indeed, in the case of some institutions several hundred beds which in normal times are reserved for the civil population are now occupied by military patients. It is true that by the use of adjoining buildings, by increasing the number of beds in some of the wards, and in other ways it has been possible in many instances to fulfil the pressing requirements of the War Office without a corresponding diminution in the number of beds retained for civilians, but when all has been said and done the fact remains that the total amount of hospital accommodation for the sick poor in London—and indeed in the whole of Great Britain—is for the time being materially less than it was before the war. Yet in spite of this it has been found, at any rate by some hospitals, that there has been no appreciable increase in the list of civil cases awaiting admission. As was noted in The Hospital of December 11 (p. 229), this somewhat unexpected experience is attributed by the Secretary of University College Hospital largely to the increased prosperity of the class of the community from which hospital patients are usually drawn; and as the same influence has been at work all over the country it is probable that although the war is directly responsible for the great strain that has been thrown on hospitals; it has indirectly brought some measure of relief—small, but sufficient to prevent the strain from becoming too great.

### How Guy's Hospital is Helping.

One of the large London hospitals—namely, Guy's—has not adopted the general policy of accepting the War Office grant for the treatment of wounded sailors and soldiers; and in taking this exceptional course the authorities were actuated by the desire to maintain at its full capacity the accommodation for civil cases. The governors took into consideration the fact that the acceptance by the other London hospitals of the Government proposals would diminish the then existing facilities for the treatment of civilians, and in their wisdom they decided that it was their duty to place themselves in a position to accept, whenever possible, civil cases for which no room could be found in other hospitals on account of the military encroachment there. They were also influenced in their decision by the circumstance that the amount of hospital accommodation on the south side of the river is relatively small. They suggested to the War Office, however, that huts might be erected in the hospital grounds for the accommodation of about a hundred and fifty beds, and made the generous offer to regard the beds, so long as funds were available, as part of Guy's Hospital, without further expense to the War Office than that involved in the erection and furnishing of the huts. While this offer was appreciated by the military authorities it was not accepted, but an opportunity occurred for the governors to help on a smaller scale. When it became known that there was a shortage of beds for officers a large ward of forty-eight beds was set aside for this purpose, and a letter was received from the King, expressing His Majesty's pleasure on hearing of the arrangements that had been made for treating so many wounded officers without the ordinary patients suffering. It should be added that no Government grant is received in respect of this accommodation. It is interesting to recall that the Stephen ward—the ward which is now in the occupation of sick and wounded officers—was thoroughly renovated eighteen months or so ago. The floor, upon a solid concrete foundation, was laid with a tile design in plantation rubber, presented by the Rubber Growers' Association. The anticipation that the demands on the hospital from the civil population would be greater than in normal times owing to the fact that a large number of beds had been allotted by other hosptals to naval and military cases has proved to be correct, and at the present time the waiting list is exceptionally heavy.

### Making Recruits Fit for Service.

The reception of civilian patients who, in the ordinary course of events, would probably have been treated at one of the other London hospitals and the allocation of a ward to wounded officers by no means exhaust the story of the work Guy's Hospital is doing in connection with the war. A ward of twenty beds has been devoted to minor surgical ailments, and as a result of treatment received there over a thousand men have up to the present been enabled to enlist for war service. Selected men who offer themselves as recruits for the Army but cannot be accepted there and then because of some physical defect which is amenable to treatment are sent to this ward so that their defects may be remedied, and in this way Guy's Hospital transforms the physically defective into serviceable recruits ready to be made into soldiers. For the extremely important work—work of great national importance—the hospital is doing in this connection no grant is received. In the majority of cases the defects consist of hernias and varicose veins; loose cartilages in the knee are also common. This kind of work is, of course, carried on at other hospitals; but we have not heard of any other voluntary hospital setting aside a ward for this special branch of military service, without accepting any grant in return from the Government. The hospital has also provided dental treatment for thousands of recruits in addition to treating wounded soldiers with fractured jaws.

### Financial Requirements of the Hospital.

In order to carry on this valuable work with as little interference as possible with the facilities provided for ordinary patients additions have been made to the available bed accommodation, and the number of beds is now not far short of seven hundred. Under normal conditions the hospital is dependent upon voluntary support to the extent of about £30,000 a year, and, in view of the present higher cost of maintenance, and of the increased demand upon the services of the charity, the Governors look for an even larger measure of financial support from the charitable public at this season, and during the present crisis, than has been given to them in the past.

